# Anthropometrical and Physiological Determinants of Laboratory and on-Snow Performance in Competitive Adolescent Cross-Country Skiers

**DOI:** 10.3389/fphys.2022.819979

**Published:** 2022-05-24

**Authors:** Ove Sollie, Thomas Losnegard

**Affiliations:** Department of Physical Performance, Norwegian School of Sport Sciences, Oslo, Norway

**Keywords:** VO_2peak_, gross efficiency, anaerobic capacity, strength, talent development, maximal aerobic power, time trial, upper-body power

## Abstract

**Purpose:** To explore the anthropometrical and physiological determinants of laboratory and on-snow performance in competitive adolescent cross-country skiers.

**Methods:** Fifty-two adolescent (25 girls) (14.8 ± 0.6 years) skiers performed an uphill treadmill rollerski session using the G2 ski skating technique. Gross efficiency (GE) was calculated from a submaximal work bout (∼84% of peak oxygen uptake; V̇O_2peak_) while V̇O_2peak_, accumulated oxygen deficit (ΣO_2def_) and laboratory performance were determined from a 3-min time trial (TT_3min_) before upper- and lower-body maximum strength were tested. Pearson’s product moment correlations and multiple regression analysis explored the relationship with anthropometrical and physiological determinations of laboratory and on-snow performance in sprint (∼1 km, ∼2.5–3 min) and distance races (5–7.5 km, ∼12–20 min) from the national championship for this age-group.

**Results:** A large correlation was found between on-snow sprint and distance performance (boys r = 0.61, girls r = 0.76, both *p* < 0.01) and for on-snow distance performance with TT _3min_ (r = 0.51 to 0.56, *p* < 0.05). V̇O_2peak_, ΣO_2def_ and GE explained ∼80% of variations in performance in the TT_3min_, but substantial lower on-snow skiing performance (∼20–30%). For the TT_3min_ performance, V̇O_2peak_ showed a very large and large correlation for boys and girls (r = 0.76 and 0.65 respectively, both *p* < 0.01), ΣO_2def_ showed a large correlation for boys and girls (r = 0.53 and 0.55 respectively, both *p* < 0.01) and age showed a large correlation for boys (r = 0.56, *p* < 0.01), with no significant correlation for girls (r = -0.19). For on-snow distance performance, V̇O_2peak_ showed a large correlation for boys (r = 0.53, *p* < 0.01) and girls (r = 0.50, *p* < 0.05). For on-snow sprint performance, upper-body strength (r = 0.55, both sexes *p* < 0.01) and body mass index (BMI) showed a large correlation for boys (r = 0.53, *p* < 0.01) and girls (r = 0.51, *p* < 0.05).

**Conclusion:** V̇O_2peak_ is an important determinant for overall XC skiing performance in competitive male and female adolescent skiers. However, upper-body strength and BMI correlate the most with sprint performance. While laboratory performance can to a large extent be explained by physiological factors, on-snow-performance for adolescents is based more on multivariate factors (tactics, equipment’s, technique, racecourse etc.), implying the need for a holistic approach to understanding the sport-specific demands in such age-groups.

## Introduction

Performance in endurance sports is mainly determined by the metabolic energy turnover (energy time^−1^) divided by the energy cost of locomotion (energy distance^−1^). Cross country (XC) skiing is a demanding and complex whole-body endurance sport that consists of races performed on undulating terrain with highly varying exercise intensity and complex interactions in energy system contributions (Gløersen et al., 2020; Sollie et al., 2020). Physiological determinants including peak oxygen uptake (V̇O_2peak_) (Sandbakk et al., 2011; Losnegard et al., 2013; Sandbakk et al., 2016), the ability to efficiently transform metabolic energy into speed (e.g., gross efficiency (GE)) (Losnegard et al., 2013)^,^ (Sandbakk et al., 2010) and the ability to repeatedly perform, and recover from, efforts above the V̇O_2peak_ (Gløersen et al., 2020; Sollie et al., 2020) are all important for XC skiing performance. Depending on the race length, sub-technique used and the technical ability of the skiers, a certain level of strength also seems necessary to optimize performance (Losnegard et al., 2019a). Moreover, differences in racing distances (durations can be between ∼3 min and ∼2 h) differentiate the magnitude of importance between these physiological determinants to some extent (Losnegard and Hallén, 2014). A substantial body of research has examined these physiological demands in (male) junior and senior XC skiers, but there are few studies of these measures in adolescent XC skiers.

Adolescent skiers (14–15 years) compete in the sprint (∼1 km, ∼2.5–3 min) and distance (5–7.5 km, ∼12–20 min) format, often on similar racecourses to those used by senior skiers. The physiological determinants of performance are possibly similar, with similar magnitudes of importance for adolescent as for senior skiers. However, anthropometric, physiological, and biomechanical differences exist between adolescent and adult skiers, and the timing of physiological development differs between adolescent boys and girls (Naughton et al., 2000). Better understanding of these determinants for adolescent skiers could provide important insights for optimizing the training process and the design of performance tests for adolescent XC skiers.

As for senior skiers, V̇O_2peak_ is a major component of performance in adolescent skiers, but growth in muscle mass, and thereby strength, appears to be a dominant factor in the increase in V̇O_2peak_ for these adolescent skiers (Landgraff et al., 2021). Moreover, anaerobic capacity and gross efficiency are also potential important determinants of performance in adolescent skiers. Greater muscle mass and strength affects anaerobic capacity (Perez-Gomez et al., 2008), and thus XC skiing performance in senior skiers (Losnegard et al., 2012a). Strength has further been shown to correlate with XC skiing performance (Stöggl et al., 2015) and may be related to improved sport-specific technique in adolescent athletes (Behringer et al., 2011). Gross efficiency (GE) is important for XC skiing performance in senior skiers three and has been found to be a discriminating factor for performance between performance levels in senior skiers (Ainegren et al., 2013). However, it is uncertain how these determinants affect adolescent skiing performance as there is little relevant research on adolescent skiers.

From an applied perspective, successful skiing performance is not affected by a single physiological determinant in isolation, but by a combination of determinants (Hébert-Losier et al., 2017; Losnegard, 2019). Previous studies have used different determinants of performance, performance levels of participants, methodology and XC skiing sub-techniques, which have explained performance differently (Mahood et al., 2001; Larsson et al., 2002; Laaksonen et al., 2020; Jones et al., 2021; Talsnes et al., 2021). A previous study of adult male skiers showed that 66% of the variation in a ∼3-min uphill skiing test could be predicted by V̇O_2peak_, anaerobic capacity and GE (Losnegard et al., 2012a). In adolescent athletes, previous research on how different physiological determinants affect performance has usually used non-specific and easily administered tests, which may not provide accurate predictions of key physiological determinants of XC skiing performance. Previous studies have explored how motor abilities and running performance (Stöggl et al., 2015) and roller ski performance (Stöggl et al., 2017) predict on-snow skiing performance in adolescent skiers, but no previous studies have used advanced laboratory measurements.

XC ski training consists of a substantial amount of on-snow and roller ski specific training (Sandbakk and Holmberg, 2014) but there is little research relating to ski-specific performance diagnostics in adolescent XC skiers. We therefore aimed to explore how age, anthropometric factors and key physiological determinants of endurance performance (i.e. V̇O_2peak_, anaerobic capacity, GE and strength) correlate with, and explain, laboratory and on-snow performance during sprint and distance competitions for adolescent male and female skiers.

## Materials and Methods

### Participants

Fifty-two adolescent competitive XC skiers (27 boys and 25 girls) participated in the study (Table 1). The athletes were recruited from local XC-ski clubs in Oslo, Norway. The inclusion criteria were experience with roller skiing and participation in national XC skiing competitions. Participants and their parents were informed of the nature of the study and the possible risks involved before giving their written consent. The study was approved by the Human Research Ethics Committee of The Norwegian School of Sport Sciences and registered with the Norwegian Centre for Research Data.

**TABLE 1:** Participants’ characteristics.

	Boys (n = 27)	Girls (n = 25)
Age (years)*	14.7 ± 0.6	14.8 ± 0.5
Body mass (kg)	57.2 ± 7.7	56.6 ± 7.8
Body height (cm)	172 ± 7	167 ± 5
BMI (kg·m2)	19.3 ± 1.6	20.3 ± 2.5
V̇O2peak (ml·kg-1·min-1)	62.1 ± 5.6	53.9 ± 5.9
Weekly training (h)**	9.3 ± 3.6	8.9 ± 3.2

Data is reported as mean ± standard deviation. *Age was calculated in weeks from the date of birth to the day of testing and converted back to years. **Weekly training was self-reported. VO_2peak_ = ski specific (roller ski) maximal oxygen uptake. VO_2peak_ was measured on the dominant side in the G2 skating technique. BMI = Body mass index

### Performance Level

The performance level was calculated as the percentage behind the mean time of the top three competitors from the national championships for this age group (“Hovedlandsrennet”, a national competition with participants from all over Norway which is held in the second half of February each year). Skiers can compete in this national race for two consecutive years (age 14 and 15) and the performance level in the present study was the skiers’ best distance and sprint (qualification round) race times from the seasons before and after testing. Both classical and skate events were used for analysis.

### Testing

All participants were tested in the pre-season period (August-September). Testing was performed in the ski skating sub-technique G2 on a roller ski treadmill (Rodby, Sodertalje, Sweden) with dimensions of 3 × 4.5 m. To exclude possible variations in rolling resistance, all skiers used the same Swenor Skate roller skis (Sport Import AS, Sarpsborg, Norway) with wheel type 1, a coefficient of friction of μ = 0.018, and Rottefella binding systems (Rottefella AS, Lier, Norway) for all tests. The coefficient of friction was measured every week during the study period and was found to be consistent throughout. All participants used Swix Triac 1.0 or 3.0 poles of a self-selected length (∼90% of body height) (Swix, Lillehammer, Norway), modified with a tip specifically adapted for use on a roller skiing treadmill. Participants were secured to the treadmill by a safety harness connected to an emergency brake during testing. Height, body mass and total mass including equipment were measured before each testing session (Seca model 877, Hamburg, Germany). V̇O_2_ was determined using a metabolic analyzer with mixing chamber (Oxycon Pro, Jaeger GmbH, Hoechberg, Germany), calibrated according to the manufacturer’s instruction manual. Heart rate (HR) was measured throughout using a Polar M400 with a 1-Hz sampling rate (Kempele, Finland). Twenty-seven skiers were tested in 2017, while the remaining skiers were tested in 2018 due to the time-consuming nature of the methods. Because a difference between breath-by-breath and mixing chamber measures was found after the first round of testing (Gløersen et al., 2020), breath-by-breath measures of V̇O_2_ were used for 27 skiers (2017) while averaged measures (mixing chamber) were used for the remaining skiers (2018). The number of skiers tested by breath-by-breath and mixing chamber measures were balanced in both boys and girls.

### Familiarization

Prior to testing, the adolescent skiers completed two sessions to familiarize them with the apparatus and the different test protocols. The first familiarization consisted of ∼35 min roller ski skating at different intensities, but if needed, the skiers could get more time to get accustomed to the treadmill. The second familiarization session consisted of a 10-min easy self-paced warm-up and two 5-min steady-state submaximal G2 work bouts with cardiorespiratory measurements, before the skiers performed a 3-min time trial (TT_3min_). Finally, familiarization with the strength testing protocol was conducted by performing the strength testing protocol (see later).

### TT_3min_

TT_3min_ was a 3-min maximal uphill time trial performed on the rollerski treadmill set to an 8° incline where the skiers were encouraged to cover as long distance as possible. The initial speed was 2.0 m s^−1^ for the girls and 2.25 m s^−1^ for the boys. This speed was fixed during the first 30 s to prevent the skiers from starting too fast. Thereafter, the skiers themselves controlled the speed by adjusting their position on the treadmill relative to laser beams situated in front of and behind them. Each contact between the front or back wheels of the skis and the lasers induced a 0.25 m s^−1^ increase or reduction in treadmill speed, respectively, conducted manually by the test leader. Visual feedback with respect to time was provided throughout. Cardiorespiratory variables (V̇O_2_ and RER) were monitored throughout the test and analyzed for V̇O_2peak,_ defined as the average of the six highest consecutive 5-s measurements (total 30 s).

### Main Test Session

The main test session included multiple submaximal work bouts with different measures, as the skiers were part of a larger research project, but only relevant work bouts and measures for the present study are included here. Following a 6-min self-paced warm-up, participants completed 5-min submaximal rollerskiing at a 6° incline and a similar estimated relative intensity (∼84% of V̇O_2peak_) and rating of perceived exertion (RPE) (RPE 15 ± 1; V̇O_2_ 84 ± 6% of V̇O_2peak_) for all skiers. Cardiorespiratory variables were monitored from 2 to 5 min and the average values were used for further analysis. RPE (Borg Scale 6-20) (Borg, 1962), was taken immediately after the work bout. The gross efficiency was calculated from this work bout.

### Calculations of Gross Efficiency (GE) and Accumulated Oxygen Deficit (ΣO_2def_)

Propulsive power on the treadmill was calculated as the sum of power against gravity and power against rolling resistance as previously described (Losnegard et al., 2012a). GE was calculated as the work rate divided by the metabolic rate under steady state conditions and ΣO_2def_ was given by subtracting the accumulated V̇O_2_ from the accumulated estimated V̇O_2_ requirements during TT_3min_ (Andersson and McGawley, 2018).

### Strength Tests

One-repetition maximum (1RM) strength tests were performed 20 min after the end of the roller ski tests using the same protocol as described by Losnegard (2011) (Losnegard et al., 2011). The order of the tests was the same for all skiers. Strength was tested separately for each arm and leg to determine whether there was a difference in strength between the right and left side. All 1RM testing was supervised by the same investigator and conducted using the same equipment, with identical equipment set-up for each skier.

*Single leg press:* The single leg press test was performed on an inclined (45°) leg press machine (Gym 2000, Vikersund, Norway). Before the test, the correct depth (90° knee angle) was measured and noted. The test started with straight legs before the skiers lowered the weights to the correct depth, at which point they received a signal from the test leader to push back up. The attempt was considered valid when the weights were returned to the starting position.

*Single arm pull-down:* The single arm pull-down was a performed on a pull-down machine (Gym 2000, Vikersund, Norway). Seating was adjusted to a 90° angle at the knees and hips, with a “neutral” spine and back resting against a backboard and both feet flat on the floor throughout the test. The “non-testing arm” rested on the opposite thigh. The pull was performed holding a custom-made ski pole grip positioned at the height of the forehead. The wire was parallel to the back support. Participants then pulled the grip straight down, with the pull defined as valid when the hand hit the bench they were sitting on in one continuous motion, without bending the torso forward away from the backboard and with both feet kept on the ground.

### Statistics

Normality of the data was assessed using the Shapiro-Wilks test (α = 0.05) and visual inspection of Q-Q plots. For statistical tests, a level of *p* ≤ 0.05 was considered significant and *p* ≤ 0.10 was considered a tendency. Figures display mean ±95% confidence interval (CI). Pearson’s product moment correlations were applied for correlations. Correlation coefficients were classified as 0.1 to 0.3 small, 0.3 to 0.5 moderate, 0.5 to 0.7 large, 0.7 to 0.9 very large and >0.9 extremely large (Hopkins, 2002). Stepwise multiple regression analyses were run with V̇O_2peak_, ΣO_2def_ and GE as independent variables to explain the TT_3min_, distance and sprint performance, separately. Boys and girls were analyzed separately, and independent *t* tests were used to compare sex differences for the different determinants. Statistical analyses were performed using Graphpad Prizm 9 (GraphPad Software, San Diego, CA) and SPSS statistical package version 24 (SPSS Inc. Chicago, IL).

## Results

### Relationship Between Laboratory and on-Snow Performance

**FIGURE 1:**
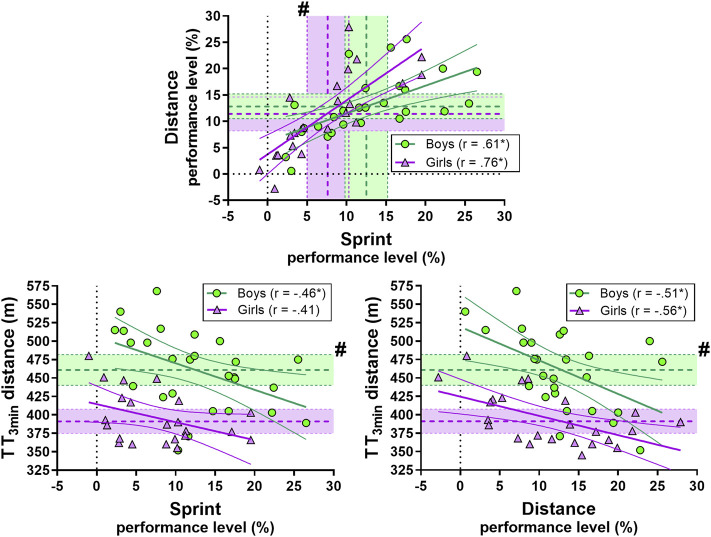
Correlation between sprint and distance performance (upper panel) and sprint and distance performance in TT_3min_ (lower panel). Green circles represent individual values for the boys. Purple triangles represent individual values for the girls. Shaded areas with dotted lines in the middle represent mean ±95% confidence interval (CI). Green is used for the boys and purple for the girls. For distance vs. sprint performance (upper panel) the horizontal shaded area represents mean ±95% for distance performance and the vertical shaded area represents mean ±95% for sprint performance. Thick and narrow lines represent the best fit regression line and the 95% confidence bands (green lines for the boys and purple for the girls). See the method section for an explanation of the performance level. * Correlation is significant at the 0.05 level. # significant difference between boys and girls.

There was a large correlation between sprint and distance performance in boys and a very large correlation in girls. Further, there was a moderate to large correlation between TT_3min_ performance and on-snow performance for both boys and girls (Figure 1).

### Multiple Regression Analysis of Performance

The full stepwise multiple regression analysis of V̇O_2peak_ relative to bodyweight (model 1), ΣO_2def_ relative to bodyweight (model 2) and GE (model 3) explained ∼80% of the variation in TT_3min_ performance in the boys and the girls (Table 2). For the sprint performance, the full model explained under 20% of the variation and was not significant in the boys or the girls (Table 3). For the distance performance, the full model explained ∼30% of the variation in the boys and the girls (Table 4).

**TABLE 2:** Full stepwise multiple regression analysis of V̇O_2peak_ (model 1), ΣO_2def_ (model 2), GE (model 3) for TT3min performance.

Model	Sex	R^2^	Change Statistics	
			R^2^ change	F statistic
1^a^	Boys	0.57	0.57	F1,24 = 32.2, *p* < 0.001
	Girls	0.42	0.42	F1,22 = 16.0, *p* < 0.001
2^a^ ^,^ ^b^	Boys	0.78	0.21	F1,23 = 22.1, *p* < 0.001
	Girls	0.57	0.15	F1,21 = 7.5, *p* = 0.012
3^a^ ^,^ ^b^ ^,^ ^c^	Boys	0.86	0.07	F1,22 = 11.1, *p* = 0.003
	Girls	0.78	0.20	F1,20 = 18.2, *p* < 0.001
Full model	Boys			F (3,22) = 43.3, *p* < 0.01
	Girls			F (2,20) = 23.2, *p* < 0.01

a V̇O_2peak_

b ΣO_2def_

c Gross efficiency

**TABLE 3:** Full stepwise multiple regression analysis of V̇O_2peak_ (model 1), ΣO_2def_ (model 2), GE (model 3) for sprint performance.

Model	Sex	R^2^	Change Statistics	
			R^2^ change	F statistic
1^a^	Boys	0.17	0.17	F1,24 = 4.8, *p* = 0.038
	Girls	0.14	0.14	F1,21 = 3.5, *p* = 0.077
2^a^ ^,^ ^b^	Boys	0.17	0.00	F1,23 = 0.0, *p* = 0.900
	Girls	0.18	0.04	F1,20 = 1.0, *p* = 0.319
3^a^ ^,^ ^b^ ^,^ ^c^	Boys	0.17	0.00	F1,22 = 0.1, *p* = 0.771
	Girls	0.19	0.01	F1,19 = 0.1, *p* = 0.749
Full model	Boys			F (3, 22) = 1.5, *p* = 0.24
	Girls			F (3, 19) = 1.5, *p* = 0.25

a V̇O_2peak_

b ΣO_2def_

c Gross efficiency

**TABLE 4:** Full stepwise multiple regression analysis of V̇O_2peak_ (model 1), ΣO_2def_ (model 2), GE (model 3) for distance performance.

Model	Sex	R^2^	Change Statistics	
			R^2^ change	F statistic
1^a^	Boys	0.28	0.28	F1,24 = 9.5, *p* = 0.005
	Girls	0.25	0.25	F1,22 = 7.4, *p* = 0.012
2^a^ ^,^ ^b^	Boys	0.30	0.02	F1,23 = 0.5, *p* = 0.470
	Girls	0.26	0.01	F1,21 = 0.2, *p* = 0.680
3^a^ ^,^ ^b^ ^,^ ^c^	Boys	0.30	0.00	F1,22 = 0.0, *p* = 0.873
	Girls	0.34	0.08	F1,20 = 2.4, *p* = 0.138
Full model	Boys			F (3,22) = 3.1, *p* = 0.05
	Girls			F (3,20) = 3.4, *p* = 0.04

a V̇O_2peak_

b ΣO_2def_

c Gross efficiency (GE)

### Determinants of Laboratory Performance (TT_3min_)

**FIGURE 2:**
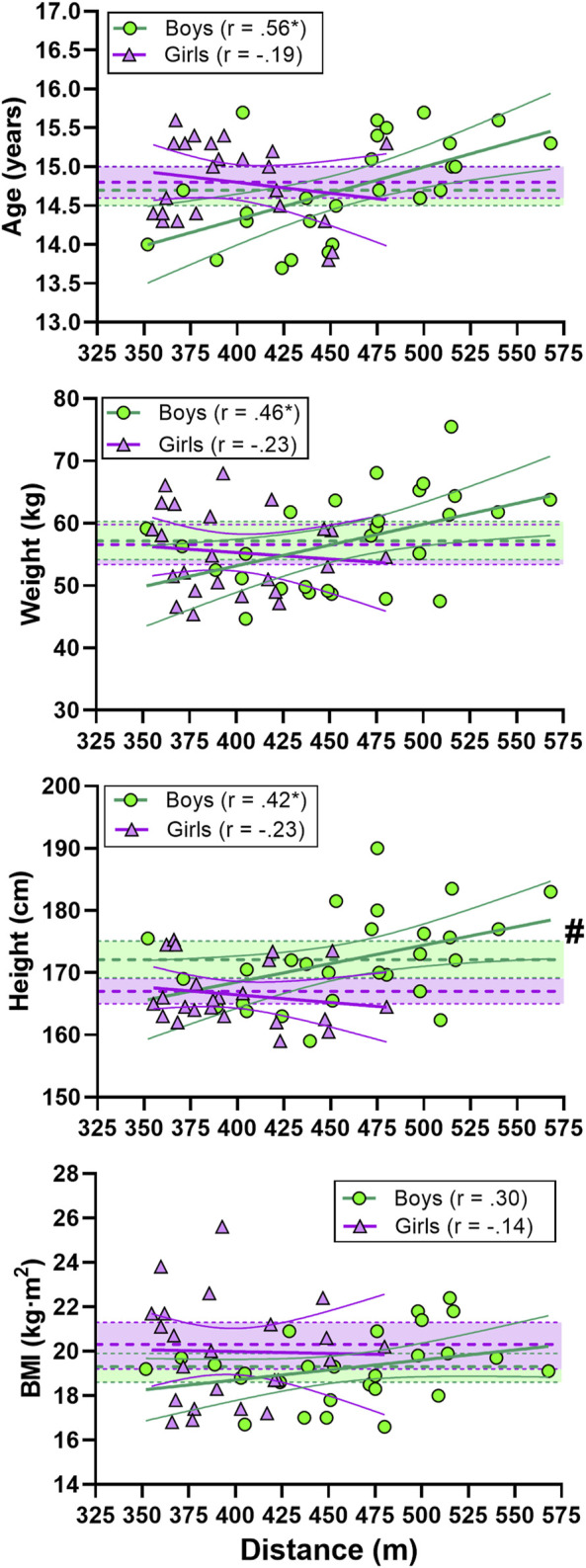
Correlations between indoor rollerski 3-min maximal uphill (8°) time-trial (TT_3min_) performance and age, weight, height and BMI. Green circles represent individual values for the boys and purple triangles represent individual values for the girls. Shaded areas with dotted lines in the middle represent mean ±95% confidence interval (CI) (green areas for the boys and purple for the girls). Thick and narrow lines represent the best fit regression lines and the 95% confidence bands (green lines for the boys and purple for the girls). * Correlation is significant at the 0.05 level. # significant difference between boys and girls.

For the boys, there was a large correlation between TT_3min_ performance and age and a moderate correlation for weight and height. No significant correlation was found for the girls (Figure 2).

**FIGURE 3:**
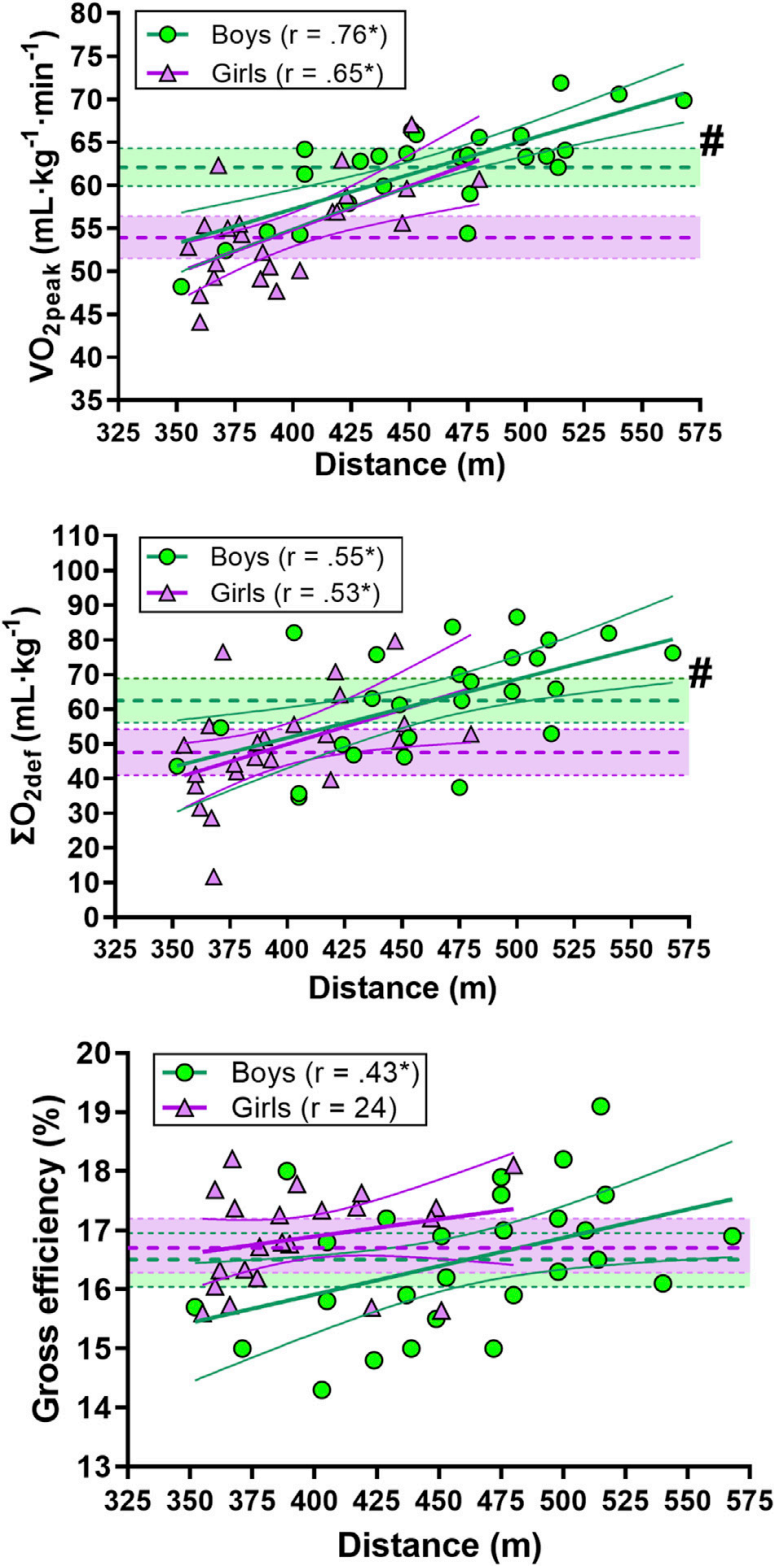
Correlation between indoor rollerski 3-min maximal uphill (8°) time-trial (TT_3min_) performance and V̇O_2peak_, ΣO_2def_ and gross efficiency (GE). Green circles represent individual values for the boys. Purple triangles represent individual values for the girls. Shaded areas with dotted lines in the middle represent mean ±95% confidence interval (CI) (green area for the boys and purple for the girls). Thick and narrow lines represent the best fit regression line and the 95% confidence bands (green lines for the boys and purple for the girls). * Correlation is significant at the 0.05 level. # significant difference between boys and girls.

There was a very large correlation between TT_3min_ performance and V̇O_2peak_ relative to body weight in boys and a large correlation in girls. There was a large correlation between TT_3min_ performance and ΣO_2def_ relative to body weight in boys and girls. Finally, there was a moderate correlation between TT_3min_ and GE in boys, with no significant correlation in girls (Figure 3).

**FIGURE 4:**
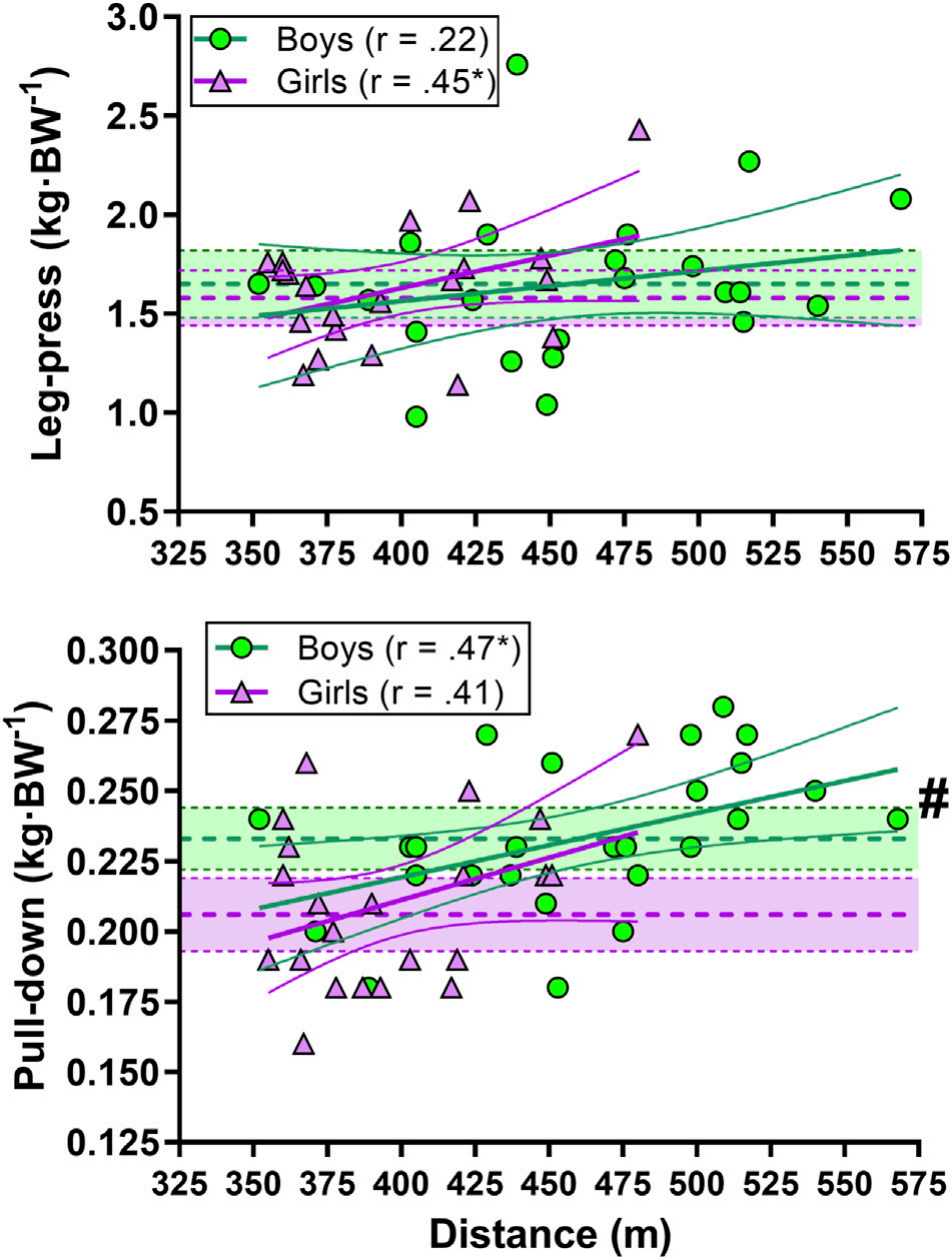
Correlation between indoor rollerski 3-min maximal uphill (8°) time-trial (TT_3min_) performance and leg-press (upper panel) and pull-down (lower panel) strength. Green circles represent individual values for the boys. Purple triangles represent individual values for the girls. Shaded areas with dotted lines in the middle represent mean ±95% confidence interval (CI) (green area for the boys and purple for the girls). Thick and narrow lines represent the best fit regression line and the 95% confidence bands (green lines for the boys and purple for the girls). * Correlation is significant at the 0.05 level. # significant difference between boys and girls.

There was a moderate correlation between TT_3min_ performance and upper-body strength in boys while a moderate correlation was found between TT_3min_ performance and leg-press strength in girls (Figure 4).

### Determinants of on-Snow Performance

**FIGURE 5:**
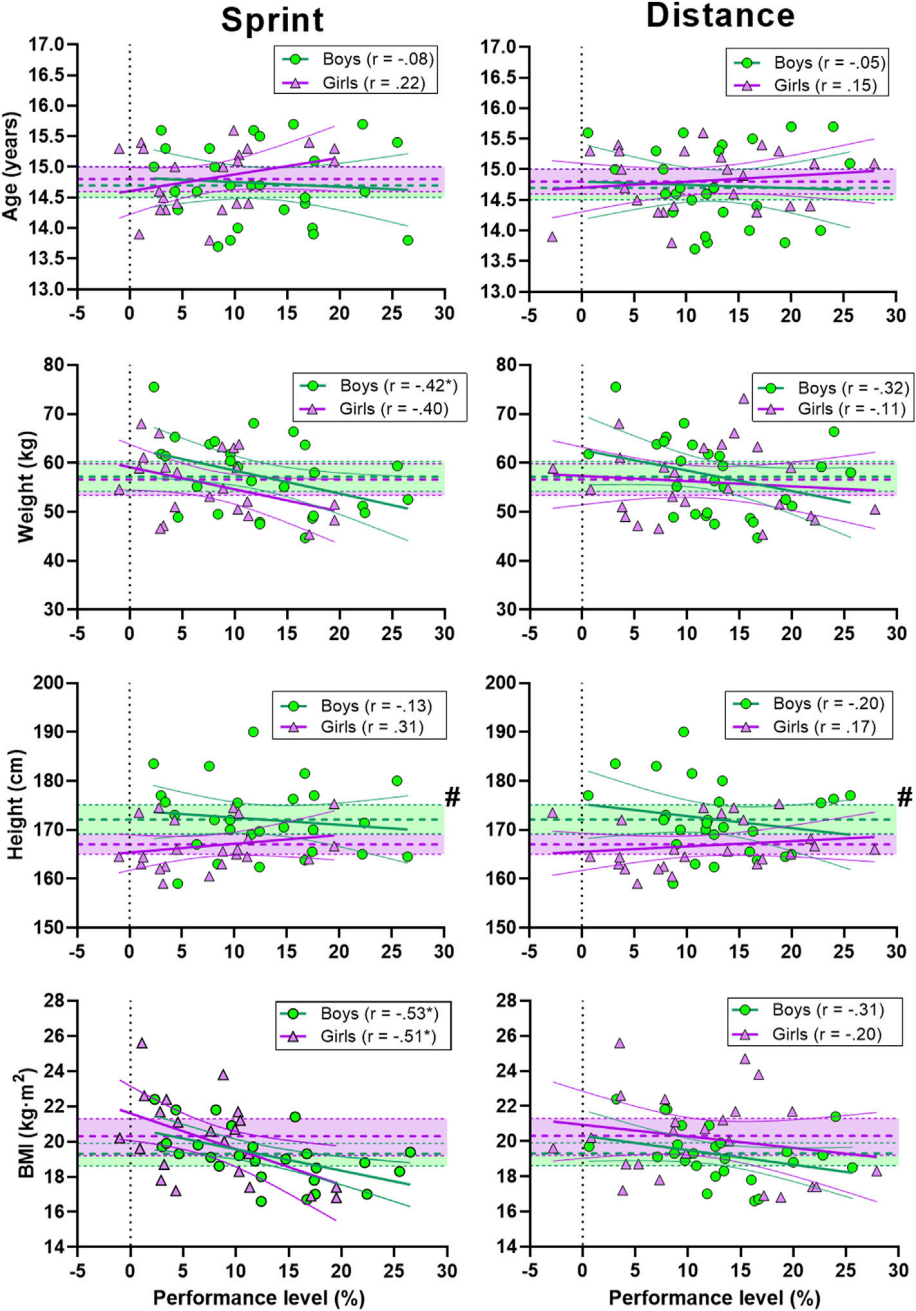
Correlation between sprint (left panel) and distance performance (right panel) and age, weight, height and BMI. Green circles represent individual values for the boys. Purple triangles represent individual values for the girls. Shaded areas with dotted lines in the middle represent mean ±95% confidence interval (CI) (green area for the boys and purple for the girls). Thick and narrow lines represent the best fit regression line and the 95% confidence bands (green lines for the boys and purple for the girls). See method section for explanation of the performance level. * Correlation is significant at the 0.05 level. # significant difference between boys and girls.

There was a moderate correlation between sprint performance and weight in boys and a large correlation between sprint performance and BMI in boys and girls. Distance performance did not significantly correlate with age, weight, height or BMI for either boys or girls (Figure 5).

**FIGURE 6:**
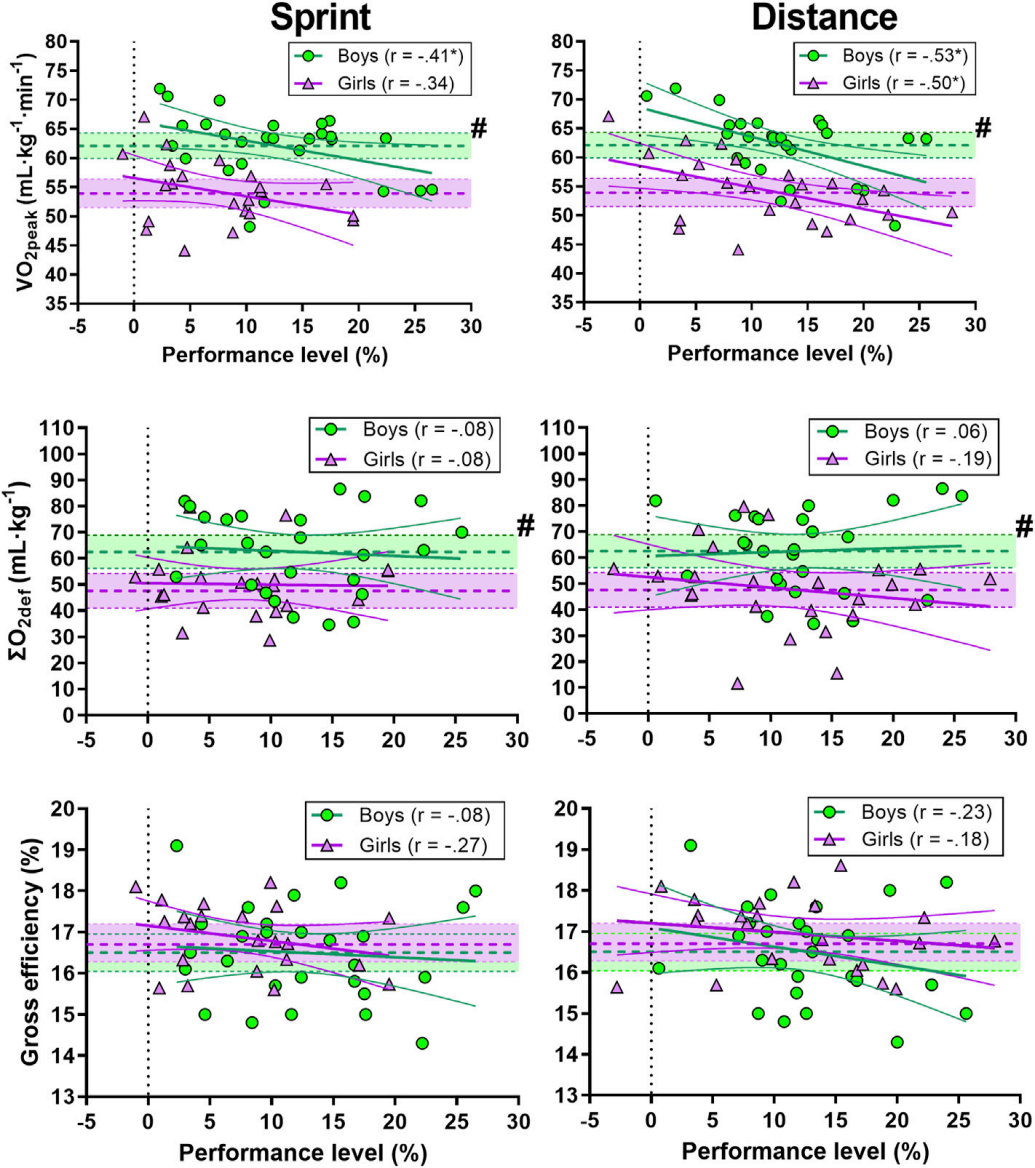
Correlation between sprint (left panel) and distance performance (right panel) and V̇O_2peak_, ΣO_2def_ and gross efficiency (GE). Green circles represent individual values for the boys. Purple triangles represent individual values for the girls. Shaded areas with dotted lines in the middle represent mean ±95% confidence interval (CI) (green area for the boys and purple for the girls). Thick and narrow lines represent the best fit regression line and the 95% confidence bands (green lines for the boys and purple for the girls). See the method section for an explanation of the performance level. * Correlation is significant at the 0.05 level. # significant difference between boys and girls.

There was a moderate correlation between sprint performance and V̇O_2peak_ relative to body weight in boys and a large correlation between distance performance and V̇O_2peak_ relative to body weight in boys and girls. Neither sprint nor distance performance was significantly correlated to ΣO_2def_ relative to body weight and GE in either boys or girls (Figure 6).

**FIGURE 7:**
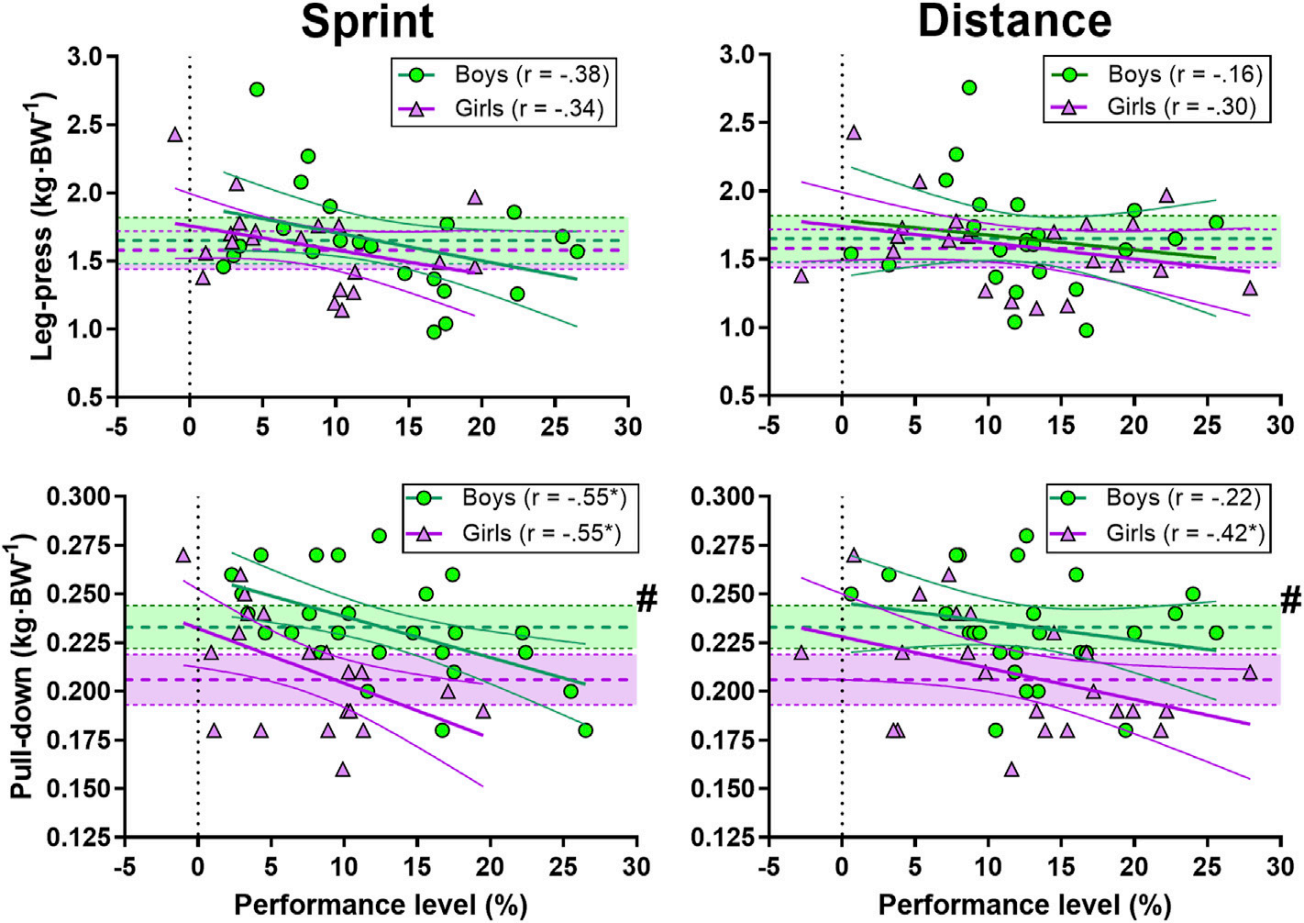
Correlation between sprint (left panel) and distance performance (right panel) and leg-press (upper panel) and pull-down (lower panel) strength. Green circles represent individual values for the boys. Purple triangles represent individual values for the girls. Shaded areas with dotted lines in the middle represent mean ±95% confidence interval (CI) (green area for the boys and purple for the girls). Thick and narrow lines represent the best fit regression line and the 95% confidence bands (green lines for the boys and purple for the girls). See the method section for an explanation of the performance level. * Correlation is significant at the 0.05 level. # significant difference between boys and girls.

There was a large correlation between sprint performance and pull-down strength in boys and girls. Further, distance performance and pull-down strength was also moderately correlated in girls (Figure 7).

Total weekly training volume had a very large correlation with TT_3min_ (r = 0.81, *p* < 0.05), and a large correlation with sprint (r = -0.53, *p* < 0.05) and distance (r = -0.56, *p* < 0.05) performance in boys (not shown in figures). No significant correlation was found in girls (r = -0.25, -0.09 and -0.16, TT_3min_, sprint and distance respectively).

## Discussion

We explored how anthropometrics and key physiological determinants of XC ski performance related to treadmill roller skiing and on-snow sprint and distance performance in competitive adolescent male and female skiers. In this study, V̇O_2peak_, ΣO_2def_ and GE explained ∼80% of the TT_3min_ performance in both sexes, which is somewhat higher than previously reported for a ∼3-min TT 12 and similar to a ∼4-min TT three in elite senior skiers. However, the present study highlights the complexity of on-snow skiing performance, where only a small part of the variation in on-snow skiing performance (∼20–30%) can be explained by V̇O_2peak_, ΣO_2def_ and GE. This discrepancy in explaining laboratory and on-snow performance is probably due to numerous factors such as the topography of the course, tactics, the quality of the equipment and weather and snow conditions, and shows that on-snow performance is based on multivariate factors and implies the need for a holistic approach for understanding the sport-specific demands in such age groups.

The large correlation between sprint and distance performance indicates that there is an overlap in determinants of performance for adolescent skiers in these two disciplines. V̇O_2peak_ relative to body weight explained a large proportion of the variation alone in the TT_3min_ and on-snow distance performance, and also contributed to sprint performance. Thus, in line with results from adult skiers (Losnegard and Hallén, 2014; Sandbakk et al., 2016), V̇O_2peak_ is an important determinant of adolescent skiing performance. The reason why some adolescents have higher V̇O_2peak_ than others is debated (Landgraff et al., 2021). A large training volume through adolescence has previously been related to a high V̇O_2peak_ (Ingjer, 1992) and increased performance (Landgraff et al., 2021). However, more specific endurance training during adolescence does not necessary produce a higher V̇O_2peak_ compared with similar volumes of training mainly aimed at developing motor skills (Landgraff et al., 2021). Our research design did not allow for detailed analysis of how training is related to performance, but it is worth noting that self-reported weekly total training hours for the boys had a large to very large correlation for the three performance settings and a large correlation with V̇O_2peak_ relative to body weight (r = 0.56). Hence, higher training volumes with a focus on developing fundamental and sport-specific motor skills (Landgraff et al., 2021) should probably be evaluated as a major part of the training in adolescent skiers.

Increased muscle mass, and thereby strength, in adolescent skiers also appears to be a dominant factor in the increase in V̇O_2peak_ (Landgraff et al., 2021) and an explanatory variable to be associated with maturity status (McNarry and Jones, 2014). We did not have any maturity measures in the present study, but increased weight in these well-trained boys most likely relates to increased growth-related muscle-mass. Further, it seemed that age and weight were more important for laboratory performance in the boys than in the girls. When ranking the skiers based on TT_3min_ performance and comparing the top and bottom ranked tertile in the boys, the top tertile were significantly older, heavier and taller compared to the bottom tertile (see appendix for methodological explanation). This was not apparent in the girls, and unlike the boys, the top-performing tertile in the girls was lighter than the bottom tertile (see appendix). This shows that boys born early in the year (relative age effect) and maturing early (i.e. greater muscle mass) had a performance advantage compared to those who were born and matured later. The relative age effect has also been previously shown in adolescent winter sport athletes (Raschner et al., 2012; Müller et al., 2017) and maturity status has previously been found to be a major confounding variable for performance in male adolescent skiers (Stöggl et al., 2015) with an estimated peak height velocity (PHV) between 13.8 ^10^ and 14.2 years (Stöggl et al., 2015). Although this is ∼5–9 months younger than the boys in the present study, this may indicate that many of the boys in the present study were in the period around PHV. Girls mature earlier than boys, and the PHV for female adolescent skiers is estimated to be ∼12.2 years (Stöggl et al., 2015; Landgraff et al., 2021). Most of the girls in the present study were thereby likely past PHV, and the top- and bottom-performing tertile were more similar in age and anthropometrics than the boys. Regardless, anthropometric variables do not seem to predict future success (de Koning et al., 19851994; Zoppirolli et al., 2020) and should thus not be an area of focus during adolescence.

Previous studies have shown a difference of importance in physiological demands for senior sprint and distance skiing 8,35, where higher muscle mass and anaerobic capacity have been found in senior sprint skiers compared to performance-matched distance skiers (Losnegard and Hallén, 2014). Similar characteristics were found for the adolescent skiers in the present study, as V̇O_2peak_ relative to body weight showed a very large (boys) and large (girls) correlation to laboratory performance and a large correlation (boys and girls) with on-snow distance performance, while upper-body strength and BMI (large correlation) contributed most to sprint performance in both sexes. Upper-body power has also previously been observed as a determinant of performance in both senior (Carlsson et al., 2012) and adolescent (Stöggl et al., 2015) skiers, probably related to the close relationship between muscle mass and anaerobic capacity 11,12. However, the large correlation found between ΣO_2def_ and TT_3min_ performance in the present study was not present in on-snow performance. The anaerobic capacity (here represented by ΣO_2def_) found in “all-out” laboratory time trials with a constant workload may not reflect how anaerobic capacity is used during real-world skiing competitions with undulating terrain and variable exercise intensity where skiers use and recover from on average “only” ∼14% (range ∼0–50%) of their ΣO_2def_ in each uphill (Gløersen et al., 2020). However, how the skiers are able to use and recover the energy reserves represented by the oxygen deficits seems important for XC skiing performance (Gløersen et al., 2020).

GE did not have a strong relationship with performance in the present study. This is in line with a recent study in senior male skiers, showing a trivial correlation between GE and outdoor performance (Talsnes et al., 2021). However, GE has previously been found to be a discriminating factor between junior and senior skiers (Ainegren et al., 2013). Furthermore, improved GE has been shown to improve XC skiing performance, highlighting that GE is very important for skiing performance (Losnegard et al., 2013)^,^ (Losnegard et al., 2019b). However, the complexity of the skiing techniques and thereby a possibly large inter-individual variation in technical solutions, together with the influence of intrinsic factors (Losnegard et al., 2012b), might diminish the discriminating effect of GE on performance in the present and other studies (Sandbakk et al., 2016; Talsnes et al., 2021).

### Limitations

We included no maturation measures in the present study and as age and anthropometrics were related to TT_3min_ and sprint performance in boys and not girls, maturity is thus a possible confounder for performance in the boys. The greater between subject variation for the boys than the girls in the TT_3min_ (Figures 2–4) might theoretically lead to larger correlations for the boys vs the girls. As both sexes were used to competing for ∼3 min, this could be due to randomness or a larger spread in the maturity status of the boys compared to the girls which may affect performance. For on-snow performance, we only assessed two races in each racing format (sprint and distance) for each skier, where we eliminated the worst performance and used the best race for further analysis. As we did not dictate which competitions the skiers participated in, these races were the only competitions all skiers participated in. A further limitation is that we performed laboratory using the G2 sub-technique, while on-snow performance was calculated using both classical and skate techniques. In addition, during XC skiing competitions, both pacing and the changing between sub-techniques may affect the performance one which we did not control for. All skiers used identical equipment during the laboratory testing, while we did not control the equipment used during on-snow racing, which might have affected the results. As adolescents develop rapidly at this age, the results may also have been affected by the relatively long time period between the laboratory testing and the on-snow races.

## Conclusion

In competitive male and female adolescent skiers there is a large (boys) and very large (girls) correlation between on-snow sprint and distance performance indicating an overlap in determinants of performance in these two disciplines. There is however a differentiation in the importance of the physiological demands for these two disciplines, as V̇O_2peak_ is the single most important determinant for distance skiing while upper-body strength and BMI are the most important determinants for sprint performance. V̇O_2peak_, ΣO_2def_ and GE explained ∼80% of the TT_3min_ performance, but only a small part of the variation in on-snow sprint and distance performance (∼20–30%). This shows that adolescent XC skiing performance is based on multivariate factors, implying the need for a holistic approach to understanding the sport-specific demands in such age-groups. Furthermore, a self-paced ski-specific TT lasting 3 min may also provide as a good predictor of XC skiing performance.

## Data Availability

The datasets generated for this study are available on request from the corresponding author.
