# Supplementary material for: Anthropometrical and Physiological Determinants of Laboratory and on-Snow Performance in Competitive Adolescent Cross-Country Skiers
Source: Front Physiol. 2022 May 24;13:819979. doi: 10.3389/fphys.2022.819979 (PMC9170917; doi:10.3389/fphys.2022.819979)
Supplement: Supplementary file 1 [file DataSheet1.docx]

Supplementary Material

Comparison of top and bottom tertiles for determinants

# Supplementary methods

The boys and girls were ranked by TT_3min_ performance. The top- and bottom-ranked tertiles (top and bottom third of the ranking) were compared using two-tailed, independent samples *t* tests for the determinants. The Holm-Šídák method was used for multiple comparisons. A level of *P* ≤ 0.05 was considered significant. Statistical analyses were performed using Graphpad Prizm 9 (GraphPad Software, San Diego, CA).

Age was calculated in weeks from the date of birth to the day of testing and converted back to years. V̇O_2peak_ and ΣO_2def_ were calculated from the TT_3min_, leg-press and pull-down were measured from a one-repetition maximum test, and gross efficiency was calculated from steady state exercise. Strength was tested separately for each arm and leg to determine whether there was a difference in strength between the right and left side. As there was no difference between right and left side, the mean between the sides is presented.

# Supplementary Tables

Table 1. Comparison of top- and bottom-ranked tertiles in boys.

| Determinants | Top tertile Mean ± SD | Bottom tertile Mean ± SD | Difference (absolute values) | *P*-value |
| --- | --- | --- | --- | --- |
| TT3_min_ performance (m) | 518 ± 23 | 402 ± 28 | 116 | < 0.01 |
| Age (years) | 15.1 ± 0.4 | 14.3 ± 0.6 | 0.8 | < 0.01 |
| Body weight (kg) | 62.4 ± 7.7 | 53.3 ± 5.3 | 9.1 | 0.01 |
| Body height (cm) | 174 ± 7 | 168 ± 4 | 6 | 0.05 |
| BMI (kg·min^2^) | 20.4 ± 1.5 | 18.8 ± 1.3 | 1.6 | 0.02 |
| V̇O_2peak_ (ml·kg^-1^·min^-1^) | 66.3 ± 3.6 | 57.7 ± 5.6 | 8.6 | < 0.01 |
| ΣO_2def_ (ml·kg^-1^) | 73 ± 10 | 51 ± 16 | 22 | < 0.01 |
| ΣO_2def_ of tot. req. (%) | 47 ± 10 | 35 ± 12 | 12 | 0.04 |
| Leg-press (kg·BW^-1^) | 1.76 ± 0.3 | 1.54 ± 0.3 | 0.22 | 0.16 |
| Pull-down (kg·BW^-1^) | 0.25 ± 0.06 | 0.22 ± 0.0 | 0.03 | < 0.01 |
| Gross efficiency (%) | 17.2 ± 0.9 | 15.9 ± 1.2 | 1.3 | 0.03 |

BMI = Body mass index; ΣO_2def_ = accumulated oxygen deficit; ΣO_2def_ of tot. req. = accumulated oxygen deficit as a percentage of total energy requirements; BW = body weight

Table2. Comparison of top- and bottom-ranked tertiles in girls.

| Determinants | Top tertile Mean ± SD | Bottom tertile Mean ± SD | Difference (absolute values) | *P*-value |
| --- | --- | --- | --- | --- |
| TT3_min_ performance (m) | 434 ± 24 | 354 ± 20 | 80 | < 0.01 |
| Age (years) | 14.6 ± 0.6 | 14.8 ± 0.5 | -0.2 | 0.57 |
| Body weight (kg) | 53.9 ± 5.7 | 61.0 ± 8.3 | -7.1 | 0.05 |
| Body height (cm) | 166 ± 6 | 170 ± 6 | -4 | 0.21 |
| BMI (kg·min^2^) | 19.6 ± 1.7 | 21.2 ± 2.6 | -1.6 | 0.13 |
| V̇O_2peak_ (ml·kg^-1^·min^-1^) | 59 ± 5 | 51 ± 6 | 8 | <0.01 |
| ΣO_2def_ (ml·kg^-1^) | 58 ± 12 | 34 ± 14 | 24 | <0.01 |
| ΣO_2def_ of tot. req. (%) | 40 ± 11 | 27 ± 12 | 13 | 0.03 |
| Leg-press (kg·BW^-1^) | 1.76 ± 0.4 | 1.50 ± 0.3 | 0.26 | 0.12 |
| Pull-down (kg·BW^-1^) | 0.22 ± 0.0 | 0.20 ± 0.0 | 0.02 | 0.28 |
| Gross efficiency (%) | 16.7 ± 1.4 | 16.7 ± 1.3 | -0.0 | 0.96 |

BMI = Body mass index; ΣO_2def_ = accumulated oxygen deficit; ΣO_2def_ of tot. req. = accumulated oxygen deficit as a percentage of total energy requirements; BW = body weight
